# Supplementary material for: Natural language generation for electronic health records
Source: NPJ Digit Med. 2018 Nov 19;1:63. doi: 10.1038/s41746-018-0070-0 (PMC6345174; doi:10.1038/s41746-018-0070-0)
Supplement: Supplementary file 1 — Supplementary Methods [file 41746_2018_70_MOESM1_ESM.pdf]

Supplementary Methods

S1. Model architecture

Like the image captioning model in Vinyals et al.[11,12], our model is designed to directly maximize the following (log) probability, where $R \in \{0,1\}^{403}$ is a sparse representation of the discrete variables in a health record and *S* is a sequence of words of length *N* associated with *R*:

$$\log p(S \mid R) = \sum_{t=0}^{N} \log(S_t \mid R, S_0, \dots, S_{t-1})$$

The encoder for our model is a simple feedforward neural network that converts the sparse record *R* into a 128-dimensional dense vector:

$$x_{-1} = W_r R$$

Because this vector is fed to the LSTM before the sequence of words, we denote its timestep as *t*=-1. We convert each word $S_t$in the sentence $S_t,\ t \in \{0 \dots N-1\}$ to a 128-dimensional dense vector x_t using a word embedding matrix W_e:

$$x_t = W_e S_t,\ \ t \in \{0 \dots N-1\}$$

For our language model, we use a single-layer RNN with a LSTM cell, which like the other layers, is 128-dimensional (biases omitted for the sake of simplicity):

$$i_t = \sigma(W_{ix} x_t + W_{im} m_{t-1})$$
$$f_t = \sigma\left(W_{fx} x_t + W_{fm} m_{t-1}\right)$$
$$o_t = \sigma(W_{ox} x_t + W_{om} m_{t-1})$$
$$c_t = f_t \odot c_{t-1} + i_t \odot h(W_{cx} x_t + W_{cm} m_{t-1})$$
$$m_t = o_t \odot h(c_t)$$
$$p_{t+1} = \text{Softmax}(m_t)$$

The input, output, and forget gates have sigmoid activations σ, and the cell state has hyperbolic tangent activations *h*. After setting the initial cell state in the LSTM to 0, we feed it the dense record vector, and then we feed it the word embeddings. Unlike Cho et al.[7], we only show the LSTM the record once, i.e. we do not concatenate it to the word embeddings at each timestep *t*.

After using an autoencoder to pretrain the weights in the encoder (mini-batch size 256; 15 epochs), we train the full model end-to-end, minimizing categorical cross-entropy loss:

$$L(R, S) = -\sum_{t=1}^{N} \log p_t(S_t)$$

S2. Explanation of modified n-gram overlap statistics

Both BLEU-N and ROUGE-N calculate scores for different values of *n*, typically from unigrams (*n*=1) up to four-grams (*n*=4); these scores are averaged to obtain the overall score for a particular sentence, and scores for sentences are averaged obtain a score for the entire corpus. As an example, BLEU calculates modified *n*-gram precision for each value of *n* in a sentence, and then takes their geometric mean as the final score, adjusting by an additional brevity penalty to encourage systems to generate longer snippets of text. There are several known issues with the metric--in particular, that it tends not to correlate as well with human judgments of translation quality as other metrics[21]--but most important for our study is that it was not designed to evaluate short translations. As an example, we can consider the following pair of chief complaints, the first being authentic and the second synthetic:

> Reference: 'cerebral infarction due to unspecified o lusion or stenosis extremity weakness stroke'
> Candidate: 'altered mental status unspecified cerebral infarction unspecified'

The sentences have some *n*-gram overlap at *n*=1 and *n*=2 and describe similar conditions, but there is no overlap at *n*=3 and *n*=4, and so the BLEU-4 score is 0.0. Smoothing functions address this discontinuity[29], but in ways that seem ad-hoc and that do not naturally transfer to ROUGE. Another issue with both metrics is that that the maximum value of *n* to consider is fixed, which creates undesirable behavior when the length of either the reference sentence or the candidate sentence is less than *n*. Here, we can consider the following pair of chief complaints, where the authentic complaint is much shorter than the synthetic complaint:

> Reference: 'heat stroke hypertension'
> Candidate: 'biba came in hospital for evaluation'

Because the reference sentence only has 3 words, it impossible for it to contain any of the 3 4-grams present in the candidate, and so evaluating BLEU at *n*=4 again leads to a score of 0. In this particular case, the score would be 0 anyway because there is no overlap at the other levels of *n*, but in general we would like to avoid penalizing a synthetic sentence simply because its corresponding authentic sentence is short. More straightforwardly, we would like our metric to make use of higher-order *n*-gram information when it is available, but only when it is available in both sentences.

To address both these issues, we propose a simple measure of *n*-gram overlap that does not require smoothing and is straightforward to calculate. The measure is calculated as follows:

1. Limit *n* to the minimum length of the 2 sentences, if either is less than *n*
2. List the unique 1-through-*n*-grams for each sentence
3. Calculate overlap (either sensitivity or PPV) for each pair of unique sentence *n*-grams
4. Average the sentence scores to obtain a single corpus score

Step 3 is equivalent to calculating the micro-average of the overlap scores for each *n*-gram level, which we find to be better suited to the variable length of the chief complaints than the weighted macro-average used in BLEU-N. Step 2 performs a similar function to the clipping term in BLEU, but because *n*-gram repetition in the chief complaints is often uninformative (consider e.g. the repetition in 'fever unspecified

fever unspecified' and 'emesis febrile seizure fever fever and vomiting and became limp'), we opt to ignore it entirely. This step also means that both measures of overlap are calculated from the same pair of *n*-gram sets, which we find to be both intuitive and appealing.

S3. Explanation of vector-space similarity metrics

We begin by noting that CIDEr is undefined when either of the sentences is shorter than the maximum value of *n* under consideration--because the magnitude for one of the TF-IDF vectors will be 0, the product of the magnitudes for both the vectors will also be 0, and cosine similarity will be undefined. We therefore modify this metric in the same was as our simplified measures of *n*-gram overlap described above by allowing *n* to vary according to the length of the sentences being compared.

We also note that BLEU, ROUGE, and CIDEr are all 0 when there is no *n*-gram overlap between sentences. Sometimes this behavior is desirable, but because the chief complaints often comprise only 1 or 2 words, it seems especially harsh (consider, e.g. that 'overdose', and 'od' would receive scores of 0 despite their clear semantic similarity). Our solution here is to take the cosine similarity between dense vector representations of the text rather than those based on *n*-grams; as long as the words in the sentences appeared in the training data for the embeddings, this measure is never undefined, and is almost always non-zero. We represent the sentence embedding as follows, where x is the one-hot vector for a single word; W_e is the word embedding matrix; m is the number of words in the sentence; and v is the average of the word embeddings appearing in the sentence:

$$v = \frac{1}{m}\sum_{i=0}^{m} W_e x_{w_i}$$

We then represent the semantic similarity between the reference sentence r and the candidate sentence c as the cosine similarity of their sentence embeddings v_r and v_c:

$$ES(r,c) = \frac{1}{N}\sum_{i=0}^{N} \frac{v_{r_i} \cdot v_{c_i}}{\| v_{r_i} \| \| v_{c_i} \|}$$

Although we did not pursue this adjustment, we note that it would be possible to combine this metric and CIDEr by applying the unigram TF-IDF weights to the word embedding matrix.

S4. Technical details about the bidirectional GRU

We implemented our chief complaint classifier as a bidirectional RNN[30] with a 200-dimensional word embedding layer, a 100-dimensional GRU as the hidden cell, and a 284-dimensional softmax layer to predict the primary CCS code for each record. With a mini-batch size of 128, the model was trained on authentic chief complaints from the training set until its loss on the validation set did not improve for 2 epochs; this occurred after 15 epochs. We again used Adam for optimization, with the learning rate set to 0.001.
